# Supplementary material for: Size and Number of Food Boluses in the Stomach after Eating Different Meals: Magnetic Resonance Imaging Insights in Healthy Humans
Source: Nutrients. 2021 Oct 16;13(10):3626. doi: 10.3390/nu13103626 (PMC8539055; doi:10.3390/nu13103626)
Supplement: Supplementary file 1 [file nutrients-13-03626-s001.zip › nutrients-1407686-supplementary.pdf]

Supplementary Materials

Table S1: Individual data on intragastric food boluses measured from *n* = 9 healthy participants for the chicken and vegetable Meal 1.

Participant	Total number of boluses	Total volume of boluses in the stomach excluding liquid (mL)	Ratio boluses volume/total stomach contents volume (%)	Mean food bolus volume (mL)	Bolus energy per unit volume (kcal/mL)	Total surface area of all food boluses (cm^2)
P101	178	166	40	0.9	1.5	691
P102	218	137	31	0.6	1.8	703
P103	388	136	35	0.4	1.8	864
P104	389	160	36	0.4	1.5	908
P105	281	126	25	0.5	1.9	706
P106	282	132	33	0.5	1.8	713
P107	159	100	25	0.6	2.4	495
P108	290	135	27	0.5	1.8	726
P109	283	156	42	0.6	1.5	809

Table S2: Individual data on intragastric food boluses measured from *n* = 9 healthy participants for the bread Meal 2.

Participant	Total number of boluses	Total volume of boluses in the stomach excluding liquid (mL)	Ratio boluses volume/total stomach contents volume (%)	Mean food bolus volume (mL)	Bolus energy per unit volume (kcal/mL)	Total surface area of all food boluses (cm^2)
P201	101	260	44	2.6	2.3	475
P202	144	315	52	2.2	1.9	792
P203	87	299	68	3.4	2.0	689
P204	106	202	54	1.9	3.0	528
P205	118	280	52	2.4	2.2	595
P206	53	344	61	6.5	1.8	508
P207	149	297	65	2.0	2.1	823
P208	81	308	67	3.8	2.0	569
P209	151	265	52	1.8	2.3	724

Table S3: Individual data on intragastric food boluses measured from *n* = 9 healthy participants for the cheese and yogurt Meal 3.

Participant	Total number of boluses	Total volume of boluses in the stomach excluding liquid (mL)	Ratio boluses volume/total stomach contents volume (%)	Mean food bolus volume (mL)	Bolus energy per unit volume (kcal/mL)	Total surface area of all food boluses (cm^2)
P301	10	117	18	12.7	3.2	223
P302	6	45	8	7.6	8.2	112
P303	6	118	21	19.6	3.2	145
P304	16	126	22	7.9	3.0	286
P305	7	122	20	17.5	3.0	181
P306	6	82	15	13.6	4.6	139
P307	10	173	28	17.3	2.2	251
P308	9	124	23	13.8	3.0	203
P309	17	84	16	4.9	4.4	216
